# Fragmented QRS in Lateral Leads on Electrocardiography Is Associated with Cardiac Dysfunction and Left Ventricular Dilation in Duchenne Muscular Dystrophy

**DOI:** 10.3390/biomedicines13040804

**Published:** 2025-03-27

**Authors:** Tetsushi Yamamoto, Shuichiro Ogawa, Yusuke Ide, Kokoro Miyazaki, Aiko Sunami, Yoshinori Nambu, Ryosuke Bo, Masafumi Matsuo, Hiroyuki Awano

**Affiliations:** 1 Department of Clinical Laboratory Science Course, Nagahama Institute of Bio-Science and Technology, Nagahama 526-0829, Japan; 2 Department of Medical Laboratory Medicine, Faculty of Health Sciences, Kobe Tokiwa University, Kobe 653-0838, Japan; 3 Department of Biophysics, Graduate School of Health Sciences, Kobe University, Kobe 657-0013, Japan; 4 Department of Pediatrics, Kobe University Graduate School of Medicine, Kobe 650-0047, Japan; 5 Graduate School of Science, Technology and Innovation, Kobe University, Kobe 657-0013, Japan; 6 Organization for Research Initiative and Promotion, Tottori University, Yonago 683-0826, Japan

**Keywords:** Duchenne muscular dystrophy, electrocardiography, cardiac dysfunction, left ventricular dilation, fragmented QRS

## Abstract

**Background/Objectives**: Duchenne muscular dystrophy (DMD) is an X-linked inherited muscle disease. Patients with DMD demonstrate improved prognosis with angiotensin-converting enzyme inhibitors and beta-blockers at the time of cardiac dysfunction. However, most deaths due to DMD are due to cardiac dysfunction. Fragmented QRS (fQRS) is an abnormal finding that forms a notch in the QRS wave on electrocardiography (ECG) and is associated with fibrosis and scarring of the myocardium. **Methods**: Patients with DMD were examined for the number of leads of fQRS, their sites of appearance, changes in cardiac dysfunction, and age using the chest leads of a synthesized 18-ECG. A retrospective analysis of 184 patients under 20 years of age with DMD and known genetic mutations was performed; they were divided into three age groups: 3–10, 11–15, and 16–20 years. The chest leads of the ECG were defined as follows: V1-3, anterior leads; V4-6, lateral leads; V7-9, posterior leads; and V3R-V5R, right-sided chest leads. Cardiac dysfunction was defined as a left ventricular (LV) ejection fraction <53% on the same day, and echocardiography was performed. LV dilation was defined as dilation beyond the normal range, considering the body surface area. **Results**: In 167 of 184 patients (91%), fQRS was present in one or more chest leads. The number of fQRS leads in the anterior and lateral walls was significantly higher in 16–20-year-olds than in 3–10-year-olds. The total number of chest leads with fQRS was 4.9 ± 3.1 in the cardiac dysfunction group and 3.5 ± 2.5 in the preserved group. The cardiac dysfunction group had a significantly greater number of fQRS leads than did the preserved group (*p* = 0.003). The group with LV dilation had a significantly greater number of fQRS leads than did the non-dilation group (*p* = 0.009). **Conclusions**: The fQRS site is associated with age, cardiac dysfunction, and LV dilation. Multivariate regression analysis revealed that the number of anterior leads of the fQRS correlated with age and that of lateral leads of the fQRS with cardiac dysfunction and LV dilation. The number of fQRS leads on the lateral wall marks cardiac dysfunction and LV dilation.

## 1. Introduction

Duchenne muscular dystrophy (DMD) is an X-linked inherited muscle disease that occurs in 1 in 5000 live birth boys [1]. DMD presents with a wide variety of complications, among which, respiratory and cardiac dysfunctions are the most common. Respiratory management with non-invasive positive pressure ventilation has improved the life expectancy of patients with DMD by an average of approximately 30 years [2,3,4]. Cardiac management of DMD is recommended using angiotensin-converting enzyme inhibitors and beta-blockers during cardiac dysfunction [5]. Consequently, the life expectancy of patients with DMD has improved. Nonetheless, cardiac dysfunction remains the leading cause of death in DMD [6].

Myocardial fibrosis precedes cardiac dysfunction in patients with DMD [7,8,9,10,11]. The frequency of fibrosis in the posterior and lateral walls is high [12], suggesting that LV fibrosis is the starting point for cardiac dysfunction. Myocardial fibrosis was assessed using cardiac magnetic resonance imaging (MRI). However, cardiac MRI presents many limitations, such as a long examination time and high invasiveness due to the use of contrast media, which limits the number of facilities that can perform the procedure. Therefore, a simple index for assessing myocardial fibrosis is necessary.

The fragmented QRS (fQRS) was first reported in 1969 as a notch waveform resulting from abnormal depolarization of the myocardium [13]. Subsequently, it was shown that the fQRS is associated with fibrosis and scarring of the myocardium [14,15,16,17,18,19,20,21]. In other words, fQRS may be a useful precursor of cardiac dysfunction in DMD. However, no reports have examined in detail the timing or site of appearance, such as the posterior leads or right ventricular side, in a large number of cases.

A standard 12-lead electrocardiography (ECG) cannot record the posterior wall and right ventricular side; nonetheless, an 18-lead ECG can record the posterior wall and right ventricular side. In this study, DMD was recorded in multiple directions using the chest leads of a synthesized 18-ECG (syn18-ECG) that computationally synthesized waveforms from a standard 12-lead ECG [22], and the number and sites of fQRS leads and their changes with cardiac dysfunction and aging were also recorded.

## 2. Materials and Methods

### 2.1. Study Design and Patient Recruitment

Patients with DMD aged 20 years or younger who visited the Department of Pediatrics, Kobe University Hospital, between July 2007 and March 2021 were included in this study. Patient data were retrospectively reviewed at the final visit. The *DMD* mutation was determined by multiplex ligation-dependent probe amplification or sequencing using genomic DNA or cDNA extracted from peripheral blood samples, as described before [23]. Patients with DMD or chromosomal abnormalities were excluded. Furthermore, patients with DMD were divided into three age groups (3–10, 11–15, and 16–20 years) to analyze age-related changes. Serum creatine kinase (CK) was measured using an automatic analyzer, JCA-BM8040 (JEOL Ltd., Tokyo, Japan), with Cygnus Auto CK (Shino-Test Corporation, Tokyo, Japan). Plasma brain natriuretic peptide was measured using the fully automated chemiluminescent enzyme immunoassay AIA-CL2400 (Eiken chemical Co., Ltd. Tokyo, Japan).

### 2.2. Electrocardiography

**Figure 1:**
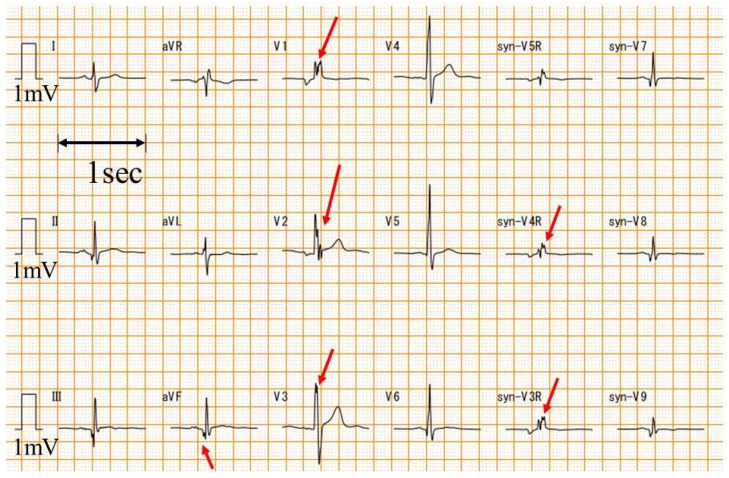
Examples of fQRS. An example of fQRS is presented. The red arrow is the fQRS. syn-: synthesized ECG was recorded with a paper speed of 25 mm/s and an amplitude of 10 mm/mV.

A standard 12-lead ECG was converted to syn18-ECG on an ECG-2550 (Nihon Kohden, Tokyo, Japan) using the logic of daming [22]. Heart rate, QRS axis, and QRS duration were automatically calculated from the ECG-2550 using ECAPS12C (Nihon Kohden, Tokyo, Japan). V1-V3, V4-V6, V7-V9, and V3R-V5R of the syn18-ECG were defined as the anterior, lateral, posterior, and right-sided chest leads, respectively. The fQRS was analyzed by one examiner (T.Y.) with extensive experience in ECG diagnosis in patients with DMD who did not know the value of LV ejection fraction (LVEF) before the fQRS analysis. The fQRS was defined as a notched QRS (Figure 1) [24].

**Figure 2:**
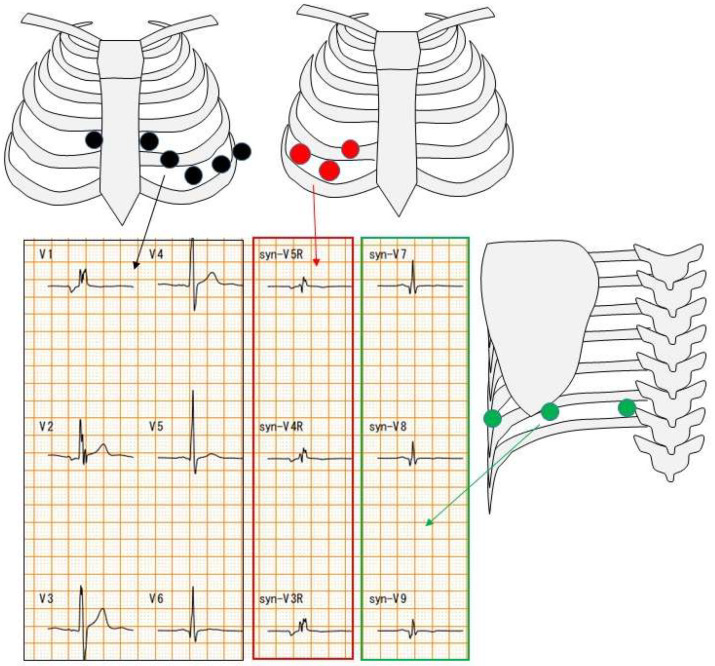
Chest electrode attachment site. Black circles indicate chest leads for 12-lead ECG; red and green circles indicate chest leads for 18-lead ECG. Red square indicates right-sided chest waveforms and green square indicates posterior waveforms.

The difference between a 12-lead ECG and an 18-lead ECG is the number of chest leads. A 12-lead ECG has six chest leads. On the other hand, an 18-lead ECG has six 12-lead ECG electrodes as well as three electrodes on the right side and three electrodes on the back (Figure 2).

### 2.3. Echocardiography

Patients were recorded in the supine position for echocardiography using Aplio XG (Canon Medical, Otawara, Japan). Echocardiographic images were obtained by recording three consecutive beats using a left parasternal and apical approach according to the recommendations of the American Society of Echocardiography [25].

LVEF was measured using the apical approach with a modified Simpson method. In the case of LVEF < 53%, DMD was defined as cardiac dysfunction [25]. LV dilation was described as an LV end-diastolic dimension above the normal range, considering the body surface area [25,26].

### 2.4. Statistical Analysis

Values are expressed as mean ± SD. The *t*-test, U-test, and Fisher test were used for two-group comparisons and the Kruskal–Wallis and Friedman tests were used for three or more group comparisons, followed by a comparison between the two groups using the Bonferroni method. Multivariate logistic regression analysis was performed to analyze independent factors associated with cardiac dysfunction and age. *p* values less than 0.05 were defined as statistically significant differences. All analyses were performed using commercially available software R version 4.1.0 (R Foundation for Statistical Computing, Vienna, Austria).

### 2.5. Ethics

This study was approved by the Ethics Committee of Kobe University (no. 1534) and Nagahama Institute of Bio-Science and Technology (no. 008).

## 3. Results

### 3.1. Patients

A total of 184 patients with DMD were included, with an age at entry of 14.0 ± 4.6 years (range, 3–20 years). Exon deletions in the DMD gene were the most common (107, 58%), followed by nonsense mutations in 38 patients (21%). Other exon duplications, small insertions/deletions, splice site mutations, and deep intron mutations were observed in 18 (10%), 16 (9%), 4 (2%), and 1 (1%) patients, respectively. LVEF at entry was 52.5 ± 12.3%.

### 3.2. Number of fQRSs

**Figure 3:**
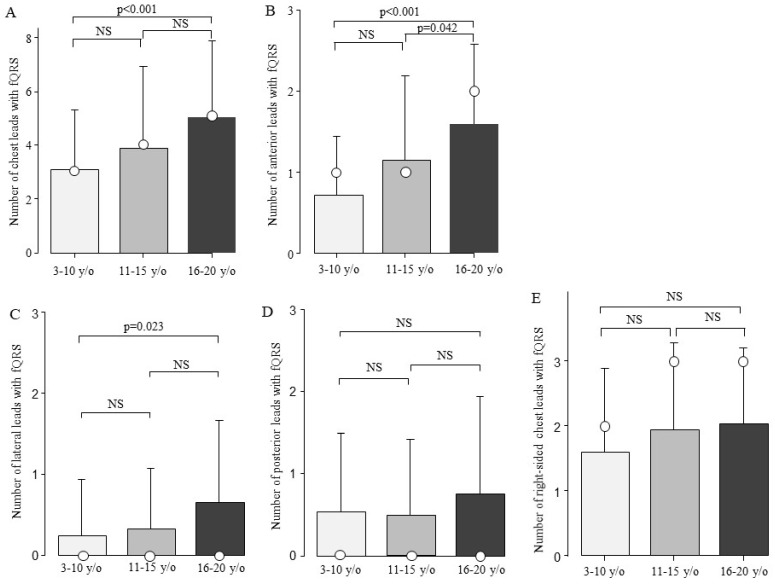
Comparison of the number of fQRS leads by age. The number of fQRS leads according to age is shown. The number of fQRS increased with age at all sites. Bars are expressed as mean ± SD. The white circles indicate the medians. (**A**) All leads; (**B**) anterior leads; (**C**) lateral leads; (**D**) posterior leads; and (**E**) right-sided chest leads. NS: Not significant.

The fQRS was present in one of the chest leads in 167 of the 184 patients (91%). fQRS was most frequently observed in right-sided chest leads (n = 139, 76%). The second most common site was the anterior leads (n = 131, 71%), followed by the posterior leads (n = 55, 30%) and lateral leads (n = 47, 26%) (Supplementary Figure S1). There were no significant differences in the type of genetic mutation, CK value, HR, QRS duration, or QRS axis between patients with and without fQRS (Table 1). The patients with fQRS were significantly older than those without fQRS (*p* = 0.003). Next, the patients with DMD were compared in terms of the number of fQRS leads among the three age groups. The total number of fQRS on the chest leads increased with age: 3.1 ± 2.2 in the 3–10-year-old group, 3.9 ± 3.1 in the 11–15-year-old group, and 5.0 ± 2.9 in the 16–20-year-old group. The total number of fQRS on the chest leads showed a significant difference between ages 3–10 and 16–20 (Figure 3A, *p* < 0.001). The DMD was examined for age-related changes at the chest lead site (Figure 3B–E). Moreover, there was a significant increase in the number of fQRS leads in the anterior and lateral walls between the ages of 3–10 and 16–20 years (Figure 3B,C). In addition, the number of anterior fQRS leads also significantly increased between the ages of 11–15 and 16–20 (Figure 3B, *p* = 0.042).

### 3.3. Relationship Between the Number of fQRS and Cardiac Function

**Figure 4:**
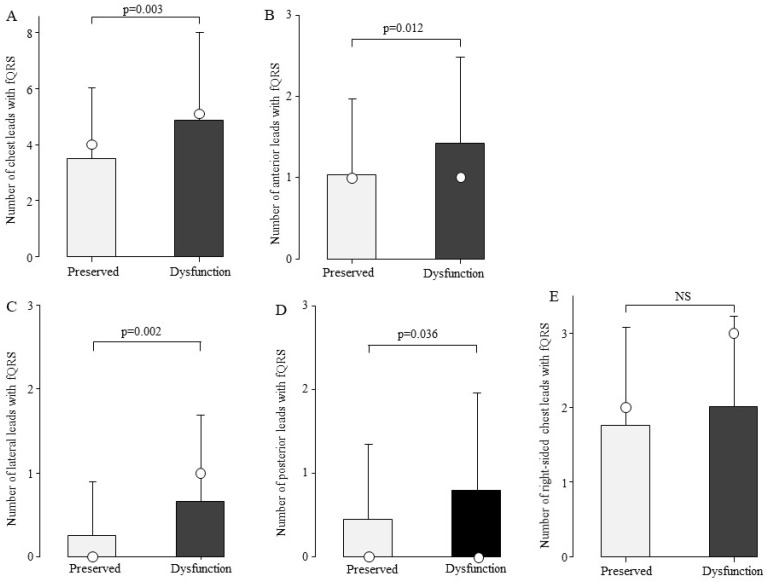
Comparison of the number of fQRS leads by cardiac function. At all sites, the number of fQRS in patients with cardiac dysfunction was greater than that in the preserved group. Bars are expressed as mean ± SD. The white circles indicate the medians. (**A**) All leads; (**B**) anterior leads; (**C**) lateral leads; (**D**) posterior leads; and (**E**) right-sided chest leads. NS: Not significant.

To examine the association between the number of fQRS and cardiac function, we compared the number of fQRS leads in the preserved cardiac function (n = 100) and cardiac dysfunction groups (n = 84). The total number of chest leads with fQRS was 4.9 ± 3.1 in the cardiac dysfunction group and 3.5 ± 2.5 in the preserved cardiac function group, with significantly more fQRS frequently in the cardiac dysfunction group (Figure 4A, *p* = 0.003). fQRS comparisons were then made by site (Figure 4B–E). The number of fQRS leads by site was significantly higher in the cardiac dysfunction group in the anterior, lateral, and posterior leads (Figure 4B–D).

### 3.4. Relationship Between the Number of fQRS and LV Dilation

**Figure 5:**
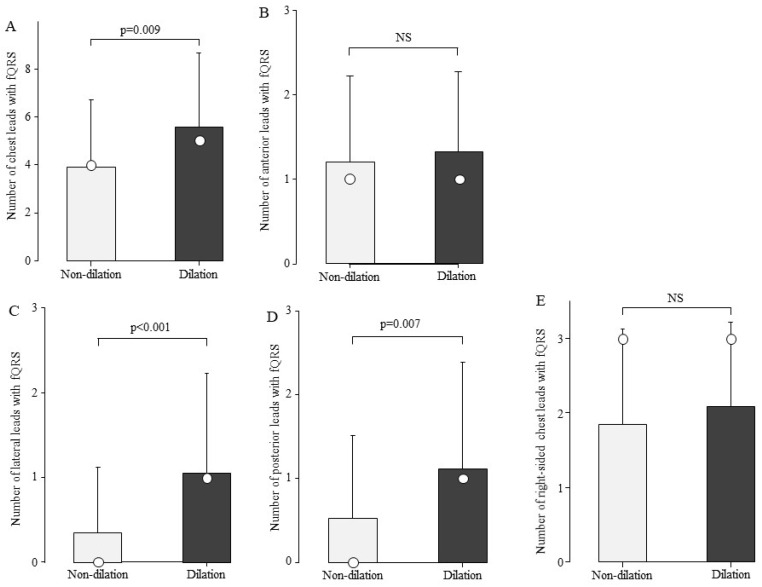
Comparison of the number of fQRS leads by left ventricular dilation. The number of fQRS in the patients in the LV dilation group was greater than that in the non-dilation group at sites other than the anterior wall. Bars are expressed as mean ± SD. The white circles indicate the medians. (**A**) All leads; (**B**) anterior leads; (**C**) lateral leads; (**D**) posterior leads; and (**E**) right-sided chest leads. NS: Not significant.

We examined the association between the number of fQRS and LV dilation. The total number of chest leads in the fQRS was 4.9 ± 3.1 in the group with LV dilation (n = 25) and 3.5 ± 2.5 in the group without LV dilation (n = 159). The group with LV dilation had a significantly higher number of fQRS leads than the group without fQRS (Figure 5A, *p* = 0.009). The fQRS was examined for its association with LV dilation by site (Figure 5B–E). In the group with LV dilatation, the number of leads with fQRS by site was significantly higher in the lateral and posterior leads (Figure 5C,D).

### 3.5. Independent Determinants Related to Cardiac Dysfunction and LV Dilation

These results indicate that the fQRS site is associated with age, cardiac function, and LV dilation. As patients with DMD have age-related cardiac dysfunction and LV dilation, fQRS may be a confounding factor for age and cardiac function. Therefore, we performed a multivariate logistic regression analysis using age and cardiac dysfunction to examine the factors associated with the number of leads in the anterior, lateral, and posterior fQRS. The number of anterior leads of the fQRS was associated with age (Table 2, *p* < 0.001) and the number of lateral leads of the fQRS was associated with cardiac dysfunction (Table 2, *p* = 0.004). Next, multivariate logistic regression analysis was performed to examine the number of lateral and posterior fQRS leads and the factors associated with LV dilation. Only the number of lateral leads was associated with LV dilation (Table 3; *p* < 0.001).

## 4. Discussion

In this study, we used syn18-ECG to examine the number of fQRS leads in patients with DMD, the number of leads by site, age changes, and their association with cardiac dysfunction. The results revealed that (1) fQRS appearing in the anterior leads was associated with age and (2) fQRS appearing in the lateral leads was associated with cardiac dysfunction and LV dilation. To our knowledge, this is the first report on the clinical implications of the fQRS site in patients with DMD.

In this study, we showed for the first time that lateral-lead fQRS was associated with cardiac dysfunction in 184 patients with DMD. Myocardial fibrosis begins in the lateral walls of patients with DMD [7]. Because fQRS is associated with myocardial fibrosis [14,15,16,17,18,19,20,21], the lateral lead fQRS was considered to reflect myocardial fibrosis, leading to cardiac dysfunction.

There have been several reports on fQRS using standard 12-lead ECG in patients with DMD. A study comparing the clinical data of 37 patients with DMD divided into two groups according to the presence or absence of fQRS reported that patients with DMD with fQRS had a lower LVEF, higher left ventricular Tei index, and more frequent ventricular arrhythmias [27]. A study of 27 patients with DMD reported that the presence of fQRS was positively correlated with age and negatively correlated with LVEF [28]. In a study comparing fQRS and MRI in 195 patients with DMD, the number of fQRS leads was higher in the group with fibrosis than in the group without fQRS [29]. The cardiac dysfunction group also had a higher number of fQRS leads than the group with preserved cardiac function. These findings suggest that the appearance of fQRS is related to cardiac dysfunction. In patients with DMD, myocardial fibrosis begins at the lateral wall on cardiac MRI [12]. Because our number of patients was larger than that in previous studies, we believe that our results are consistent with cardiac MRI results examining myocardial fibrosis. By site, this is the first study to show that the lateral wall is associated with cardiac function and dilation. This finding is consistent with the fact that myocardial fibrosis begins in the lateral wall on MRI. Lateral-lead fQRS may reflect myocardial fibrosis.

In this study, syn18-lead was used. A standard 12-lead ECG makes it challenging to assess the posterior wall, which is the site of cardiac dysfunction in DMD. Therefore, we used syn18-lead to evaluate the posterior wall in patients with DMD. However, only the lateral leads showed a significant association between cardiac dysfunction and LV dilation. In contrast, the right-sided chest-lead and posterior-lead fQRS did not show a significant association between cardiac dysfunction and LV dilation. In other words, fQRS associated with cardiac dysfunction or LV dilation can be determined using a standard 12-lead ECG.

This study found that the number of anterior leads in fQRS was associated with age. No studies have been conducted on myocardial fibrosis of the right ventricle in patients with DMD. The right ventricle may be more prone to myocardial fibrosis because the right ventricular wall is thinner than the LV wall [30]. Therefore, fQRS is likely the most common condition in right-sided chest leads. There are only three right-sided chest leads, and the number of fQRS can only be in a narrow range of only four levels (0–3). In the present results, the 11–15 and 16–20 age groups showed a median of 3 (Figure 3E), with no measurable age change beyond 11 years. The reason why the association between age and number was not apparent may have been influenced by the narrow assessment range. In arrhythmogenic right ventricular cardiomyopathy, epsilon waves, which represent conduction disturbances due to fatty degeneration of the myocardium [31], also appear and are detected in the anterior leads [32,33]. Thus, the anterior leads may exhibit an ECG signal that reflects the right ventricle.

On echocardiography, we expected the posterior leads fQRS to be related to cardiac function because asynergy begins in the LV posterior wall [34,35,36,37]; however, no association was found. This may have been because the posterior leads were farther from the myocardium than the lateral leads and may not have adequately captured the changes. Multivariate regression analysis revealed that posterior leads tended to be associated with cardiac dysfunction (*p* = 0.058), which requires further investigation.

The fQRS of V4-6 was considered a surrogate indicator of myocardial fibrosis associated with cardiac dysfunction. The recording of V4-V6 is possible with a 12-lead ECG, which can be performed in many hospitals. Thus, fQRS can be used to assess myocardial fibrosis in many hospitals. Furthermore, the fQRS is determined by the shape of the QRS on electrocardiography; therefore, there is no need for measurement or other time-consuming procedures. Therefore, the fQRS is considered useful in the management of myocardial damage in DMD because it is an easy marker to record and evaluate.

This study has several limitations. First, because this was a single-center study, a patient selection bias may have existed. In the future, it will be necessary to verify these findings in many hospitals. Second, because this was a cross-sectional study, it was not possible to compare the timing of fQRS onset with that of cardiac dysfunction. Therefore, it remains unclear whether the appearance of fQRS precedes or results in cardiac dysfunction. Further studies with different research designs are required to clarify this issue. The syn18-lead ECG showed no chest electrodes on the inferior wall; therefore, the inferior wall was not evaluated in this study.

## 5. Conclusions

Lateral-lead fQRS was associated with cardiac dysfunction and LV dilation.

## Figures and Tables

**Table 1:** Comparison of fQRS.

		With fQRS	Without fQRS	*p* Value
Number of patients		167	17	
Age (years)		14.3 ± 4.6	10.6 ± 4.1	0.003
Creatine kinase (IU/L)		3791.5 ± 3974.2	8486.6 ± 13,346	0.21
Brain natriuretic peptide (pg/mL)		21.5 ± 40.6	11.6 ± 11.3	0.036
Echocardiographic parameters				
	Left ventricular end-diastolic dimension (mm)	42.8 ± 8.9	38.3 ± 3.7	<0.001
	Left ventricular end-systolic dimension (mm)	32.2 ± 10.8	25.9 ± 4.8	<0.001
	Left ventricular ejection fraction (%)	51.8 ± 12.3	60.4 ± 8.4	0.001
Electrocardiographic parameters				
	Heart rate (BPM)	85.7 ± 14.4	91.9 ± 14	0.11
	QRS duration (msec)	92.8 ± 12.7	86.3 ± 11.9	0.052
	QRS axial (degree)	74.4 ± 39.7	75.8 ± 17.2	0.80
Mutation type				
	Deletion	106	1	
	Nonsense mutation	36	2	
	Duplication	17	1	
	Small insertion/deletion	16	0	
	Splice site mutation	4	0	
	Deep intron mutation	1	0	0.30

**Table 2:** Multivariate logistic analysis of fQRS with cardiac dysfunction and age.

Position	Variable	Odds Ratio	95% Confidence Interval	*p* Value
Anterior	Age	1.14	1.06–1.23	<0.001
	Dysfunction	0.816	0.329–2.02	0.66
Lateral	Age	1.06	0.961–1.17	0.24
	Dysfunction	2.71	1.37–5.39	0.004
Posterior	Dysfunction	1.85	0.979–3.51	0.058

**Table 3:** Multivariate logistic analysis of fQRS with LV dilation.

Variable	Odds Ratio	95% Confidence Interval	*p* Value
Lateral	2.00	1.35–2.95	<0.001
Posterior	1.15	0.725–1.82	0.55

## Data Availability

The original contributions presented in the study are included in the article, further inquiries can be directed to the corresponding author.

## Supplementary Materials

The following supporting information can be downloaded at https://www.mdpi.com/article/10.3390/biomedicines13040804/s1, Figure S1: Comparison of fQRS leads by site. The number of fQRS was significantly greater in the right-sided chest leads than at other sites. Bars are expressed as mean ± SD. White open circle shows the median.

## Abbreviations

BPM	Beats Per Minute
CK	Creatine Kinase
DMD	Duchenne Muscular Dystrophy
ECG	Electrocardiography
fQRS	Fragmented QRS
HR	Heart Rate
IU/L	International Units per Liter
LV	Left Ventricle
LVEF	Left Ventricular Ejection Fraction
MRI	Magnetic Resonance Imaging
SD	Standard Deviation
