# Supplementary material for: Fragmented QRS in Lateral Leads on Electrocardiography Is Associated with Cardiac Dysfunction and Left Ventricular Dilation in Duchenne Muscular Dystrophy
Source: Biomedicines. 2025 Mar 27;13(4):804. doi: 10.3390/biomedicines13040804 (PMC12024901; doi:10.3390/biomedicines13040804)
Supplement: Supplementary file 1 [file biomedicines-13-00804-s001.zip › biomedicines-3541248-supplementary.pdf]

Supplementary figure

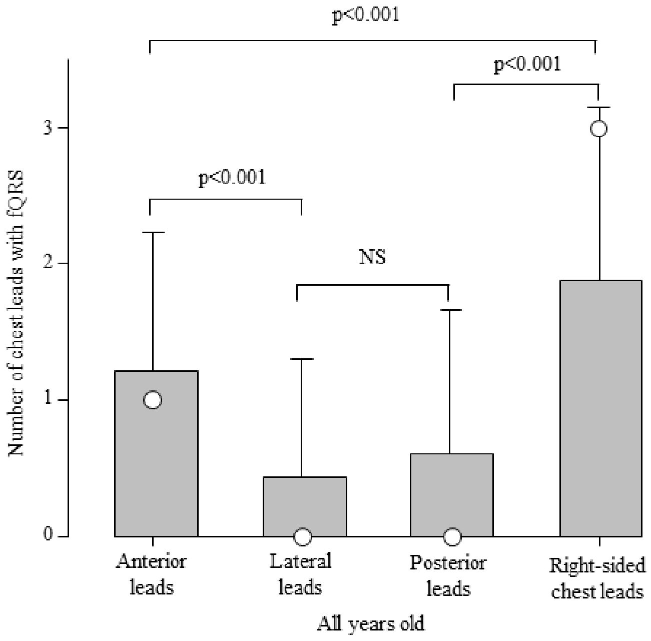

Figure S1. Comparison of fQRS leads by site.
The number of fQRS is significantly greater in the Right-sided chest leads than in other sites.
Bars are expressed as mean ± SD. White open circle show median.
